# Low Lipoprotein(a) Concentration Is Associated with Cancer and All-Cause Deaths: A Population-Based Cohort Study (The JMS Cohort Study)

**DOI:** 10.1371/journal.pone.0031954

**Published:** 2012-04-02

**Authors:** Motoji Sawabe, Noriko Tanaka, Makiko Naka Mieno, Shizukiyo Ishikawa, Kazunori Kayaba, Ken-ichi Nakahara, Satoru Matsushita, J. M. S. Cohort Study Group

**Affiliations:** 1 Bioresource Center for Geriatric Research, Tokyo Metropolitan Geriatric Hospital, Tokyo, Japan; 2 Department of Internal Medicine, Tokyo Metropolitan Geriatric Hospital, Tokyo, Japan; 3 Department of Biostatistics, Harvard School of Public Health, Boston, Massachusetts, United States of America; 4 Department of Medical Informatics, Jichi Medical University, Shimotsuke, Japan; 5 Center for Community Medicine, Jichi Medical University, Shimotsuke, Japan; Universidad Peruana Cayetano Heredia, Peru

## Abstract

**Background:**

Experimental studies support the anti-neoplastic effect of apo(a), but several clinical studies have reported contradictory results. The purpose of this study was to determine whether a low lipoprotein(a) [Lp(a)] concentration is related to mortality from major causes of death, especially cancer.

**Methods:**

The subjects were 10,413 participants (4,005 men and 6,408 women) from a multi-center population-based cohort study in Japan (The Jichi Medical School cohort study). The average age at registration was 55.0 years, and the median observation period was 4,559 days. As the estimated hazard ratio was high for both the low and very high Lp(a) levels, we defined two Lp(a) groups: a low Lp(a) group [Lp(a)<80 mg/L] and an intermediate-to-high Lp(a) group [Lp(a)≥80]. Participants who died from malignant neoplasms (n = 316), cardiovascular disease (202), or other causes (312) during the observation period were examined.

**Results:**

Cumulative incidence plots showed higher cumulative death rates for the low Lp(a) group than for the intermediate-to-high Lp(a) group for all-cause, cancer, and miscellaneous-cause deaths (p<0.001, p = 0.03, and p = 0.03, respectively). Cox proportional hazards analyses with the sex and age of the participants, body mass index, and smoking and drinking histories as covariates showed that a low Lp(a) level was a significant risk for all-cause, cancer, and miscellaneous-cause deaths (p<0.001, p = 0.003, and p = 0.01, respectively). The hazard ratio (95% CI) [1.48, 1.15–1.92] of a low Lp(a) level for cancer deaths was almost the same as that for a male sex (1.46, 1.00–2.13).

**Conclusions:**

This is the first report to describe the association between a low Lp(a) level and all-cause or cancer death, supporting the anti-neoplastic effect of Lp(a). Further epidemiological studies are needed to confirm the present results.

## Introduction

Large-scale prospective cohort studies and their meta-analyses, including our study, have shown that hyperlipoproteinemia(a) is a risk factor for coronary artery disease and stroke [1]–[4]. To reduce the risk of hyperlipoproteinemia(a), the development of Lp(a)-lowering therapies has been pursued, including lipid apheresis and the use of antisense oligonucleotide [5]–[7].

Meanwhile, apolipoprotein(a) [apo(a)] is a unique protein found only in old-world primates, including human beings and hedgehogs. Despite the established association between Lp(a) and cardiovascular disease, the physiological function and the metabolism of apo(a) and the association of apo(a) with other diseases remain unknown. The apo(a) gene (*LPA*) and the plasminogen gene share a number of characteristic repeated domains called Kringle. Angiostatin, a degraded product of plasminogen, exerts an anti-neoplastic effect by inhibiting angiogenesis [8]. A phase II study on angiostatin has been performed in patients with non-small cell lung cancer [9]. As *LPA* also has Kringle structures, apo(a) may also have an anti-neoplastic effect [10]. A recombinant protein (LK68) of *LPA* Kringle type IV and V experimentally suppressed tumor growth and capillary density within tumors in mice [11]. Gene therapy inducing an LK68 recombinant gene suppressed the tumor growth of transplanted hepatocellular carcinoma in mice [12] and liver metastasis and peritoneal dissemination in a murine colon cancer model [13], [14]. Tumor growth and angiogenesis were also suppressed in apo(a)-transgenic mice [15]. An 11-amino acid short peptide deduced from *LPA* Kringle type V also had an anti-neoplastic effect [16]. All these experimental studies support the anti-neoplastic effect of apo(a), but several clinical studies have reported contradictory results, with the serum Lp(a) level being elevated in cancer-bearing patients or not being significantly different from that of the control group [17]–[25]. These clinical studies, however, had several limitations. The number of cancer cases was generally small, and some studies lacked data regarding the histological type or the clinical stage of the cancer or the presence or absence of metastasis to the liver, which produces Lp(a). No prospective studies regarding the association between Lp(a) and cancer have been reported to date.

To test the hypothesis that a low Lp(a) concentration is related to cancer deaths, we analyzed data from the Jichi Medical School (JMS) cohort study, a large-scale, multi-center, population-based cohort study conducted in Japan. To our surprise, a low Lp(a) concentration was associated not only with cancer deaths, but also with all-cause and miscellaneous-cause deaths. The implications of these interesting results are also discussed in this article.

## Methods

### Ethics Statement

The Jichi Medical University ethics committee approved the study, and each subject provided their written informed consent.

### The JMS Cohort Study

The JMS cohort study was comprised of 12 population-based cohorts from the Tohoku to Kyusyu regions in Japan; the study was started in 1992 to clarify the risk factors for cardiovascular and cerebrovascular diseases among the Japanese population. Local residents attending regular medical checkups based on a legal mass-screening system were asked to participate in the JMS cohort study [26]. A total of 12,490 residents were registered between 1992 and 1995. These residents included 95 participants who did not consent to the follow-up studies and 2 participants who moved away prior to the baseline study. All the participants were Japanese according to the information obtained from their residence certificates. In 10,692 of the participants (85.6%), the serum Lp(a) level was measured at the time of the baseline examination using an ELISA kit (Biopool, Uppsala, Sweden; interassay CV, 3.5) [27], [28]. The lowest detection limit was 10 mg/L, and the undetectable level was considered to be 5 mg/L for the statistical analysis. The main reason for the relatively high percentage (14.4%) of Lp(a)-unmeasured subjects was the lack of an Lp(a) measurement kit in some areas. There were no meaningful differences between the Lp(a)-unmeasured and the Lp(a)-measured subjects. A survival analysis showed no significant difference between the two groups (p = 0.66). The subjects of this study were generally healthy and consisted of 10,413 participants, excluding 279 participants with a history of stroke, myocardial infarction, or malignant neoplasm at the time of the baseline examination.

The first quartile, median, and third quartile of the observation period were 4,201 days, 4,559 days, and 4,900 days, respectively. More than 90% of the participants were followed for more than 3,837 days.

### Habits, medical history, and diagnostic criteria

The habits and medical history of each participant were obtained using a questionnaire administered at the time of the baseline examination [26]. The smoking history covered both past and current smoking habits. If a participant consumed alcohol more than three times a week, he or she was considered to be a habitual drinker. The drinking history covered both past and current drinking habits. The diagnostic criteria for myocardial infarction defined in the World Health Organization’s MONICA project and the diagnostic criteria for stroke defined by the Yanagawa group for stroke research of the Japanese Ministry of Health and Welfare were adopted.

### Death of the participants

Overall, 830 participants died during the observation period; the cause of death was obtained from each participant’s death certificate. The participants died from malignant neoplasms (316 participants), cerebrovascular disease (97), heart disease (92), miscellaneous vascular disease (13), other diseases or causes (311), and an unknown cause (1). The primary sites of the malignant neoplasms were as follows: lung (59), stomach (28), colon (19), liver (18), biliary tree (23), pancreas (21), hematopoietic organs (21), and other organs (127).

### Statistical analysis

**Figure 1:**
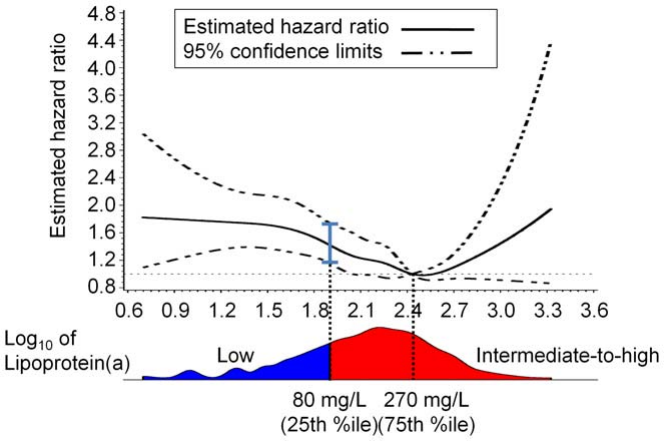
Estimated hazard ratio of death against lipoprotein(a) level. Note a U-shaped curve of the estimated hazard ratio, especially the significant increase in the hazard ratio for lipoprotein(a) levels below 80 mg/L. The reference value for lipoprotein(a) was set at 270 mg/L.

To assess the hypothesis that a low Lp(a) concentration is related to cancer deaths, we first examined the possible non-linear relation between the Lp(a) level and the survival time, such as the hazard ratio for the low Lp(a) concentration non-parametrically assessed using restricted cubic splines [29]. We used the likelihood ratio test for non-linearity, comparing the model only with the linear term and that with the linear and cubic spline terms. Setting the Lp(a) threshold at 80 mg/L seemed reasonable, considering the U-shaped curve of the estimated hazard ratio versus the Lp(a) level shown in Figure 1. Based on the results of this analysis and considering the hazard ratio and the number of cases in each group, we defined the two Lp(a) groups as follows: a low Lp(a) group [Lp(a)<80 mg/L (25th percentile); n = 2,537], and an intermediate-to-high Lp(a) group [Lp(a)≥80 mg/L; n = 7,876]. The Fisher exact test or Mann-Whitney test was used to assess the statistical significance of differences in categorical variables or continuous variables, respectively, between the groups. For each group, cumulative incidence curves were used in a competing-risk setting to calculate the probability of cardiovascular, cancer, and miscellaneous-cause mortality for two Lp(a) groups, since we confirmed that the type-specific hazard functions were not the same for all event types (Figures S1 and S2). The Gray test was used for group comparisons of cumulative incidence [30]. The overall survival was calculated using the Kaplan-Meier method, and the log-rank test was used for group comparisons of overall survival. The association of Lp(a) with the outcomes was evaluated using multivariate analyses, the use of a multivariate Cox proportional-hazards regression to adjust for other outcomes, and the use of the Fine and Gray proportional hazards model for the subdistribution of a competing risk for other outcomes [31]. All the models were adjusted for age, sex, body mass index, and smoking and habitual drinking histories. To confirm the feasibility of this two grouping in cause-specific deaths, we performed a Cox proportional hazard analysis of the serum lipoprotein(a) levels divided into quartiles. Setting the Lp(a) threshold at 80 mg/L seemed to be reasonable for cause-specific deaths (Table S1). The power of the analysis was calculated based on a log-rank test to detect a hazard ratio of 1.4 for the endpoint between the low Lp(a) group and the intermediate-to-high Lp(a) group at a two-sided overall significance level of 5%. Allowing for a loss-to-follow-up rate of less than 1% for all groups, a 98.5% power was estimated for a 12.5-year follow-up.

All the p values were two-sided, and a p value less than 0.05 was considered significant. The statistical analysis was performed using the SAS system for Windows (ver. 9.1.3; SAS Institute Inc, Cary, NC, USA). The cause-specific cumulative death rate was calculated using R2.13.1.

## Results

### Serum Lp(a) level

The Lp(a) level ranged from 5 mg/L to 2,150 mg/L and showed a highly skewed distribution toward the lower levels. The 25th, 50th, and 75th percentiles were 80, 150, and 270 mg/L, respectively. The log-transformed Lp(a) values followed a normal distribution (p<0.05).

As mentioned in the Methods section, we assessed the non-linear relation between the Lp(a) level and the survival time. Figure 1 shows a U-shaped curve for the estimated hazard ratio against the Lp(a) level; the hazard ratio gradually decreased with the Lp(a) level from 0 to 270 mg/L and sharply increased thereafter. The shape of the curves did not depend on gender, and the Lp(a) thresholds for both genders were the same as that for all the participants.

Table 1 summarizes the baseline data of the subjects according to two Lp(a) groups. The low Lp(a) group was characterized by a male predominance; a younger age at registration; more frequent smoking and alcohol drinking habits; a larger height, weight, and body mass index; a higher total cholesterol level; and a lower triglyceride level.

### Non-parametric survival analysis

**Figure 2:**
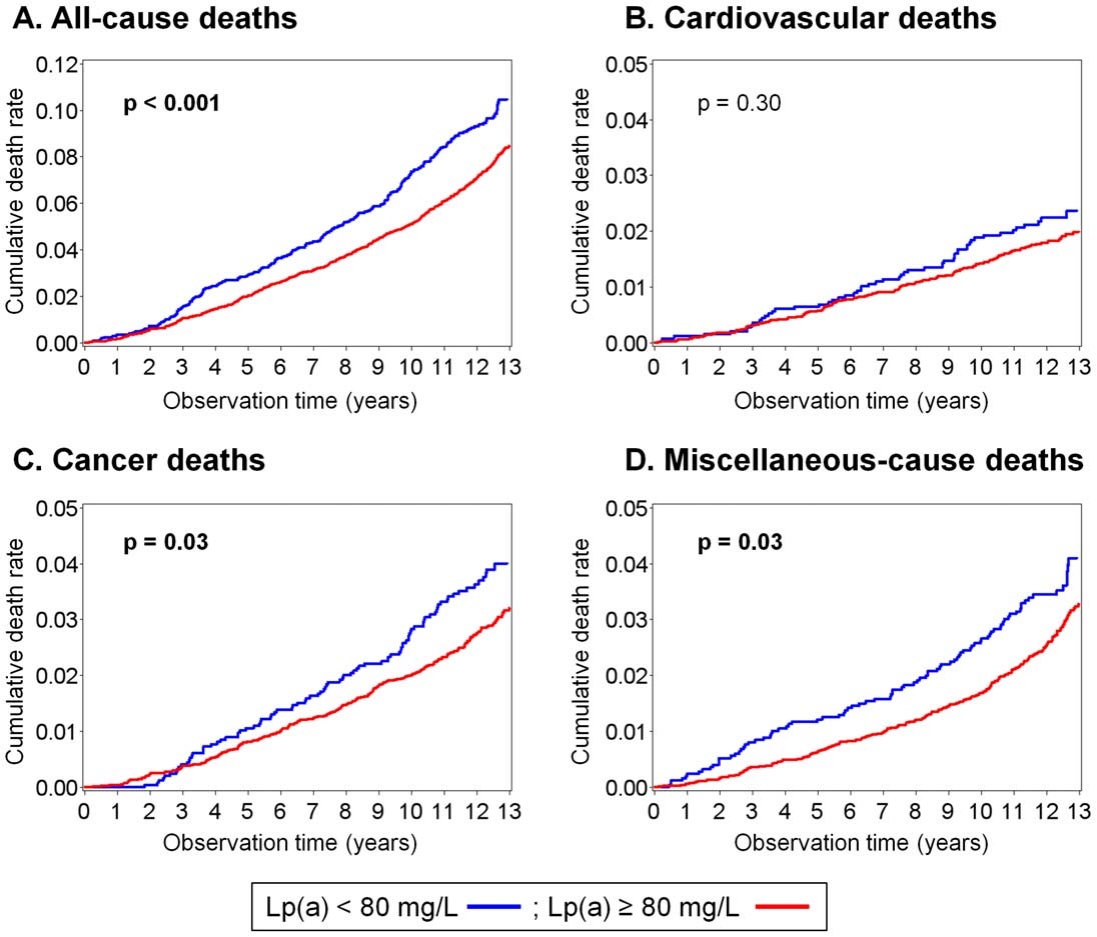
Cumulative death rates for all-cause and cause-specific deaths among two lipoprotein(a) [Lp(a)] groups. The cumulative death rates of the low Lp(a) group were significantly higher than those of the intermediate-to-high Lp(a) group for all-cause, cancer, and miscellaneous-cause deaths.

The survival analysis showed higher cumulative death rates for the low Lp(a) group than for the intermediate-to-high Lp(a) group for all-cause death, cancer death, and miscellaneous-cause death, as shown in Figure 2. The subjects were divided into three Lp(a) groups: a low Lp(a) group [Lp(a)<80 mg/L (25th percentile); n = 2,537], an intermediate Lp(a) group [80≤Lp(a)<550 mg/L (95th percentile); n = 7,332], and a very high Lp(a) group [Lp(a)≥550 mg/L; n = 544]. The cumulative death rate of the very high Lp(a) group was not higher than that of the intermediate Lp(a) for all-cause or any cause-specific deaths (Figure S3).

Additionally, we estimated the survival curves of the primary-site-specific cancer deaths (Figure S4). In the low Lp(a) group, the cumulative death rates were significantly higher for liver cancer death (n = 18). The cumulative death rates were not significantly different between the low and intermediate-to-high Lp(a) groups for deaths caused by digestive system cancers excluding liver cancer (n = 104), lung cancer (n = 59), and other cancers (n = 135).

**Table 1:** Baseline characteristics of the study cohort.

	Total		Low Lp(a) group*		Intermediate to high Lp(a) group*		
	No. of cases	Mean ± SD or No. of cases(%)	No. of cases	Mean ± SD or No. of cases(%)	No. of cases	Mean ± SD or No. of cases(%)	p value†
Male sex (%)	10,413	4,005 (38.5%)	2,537	1,113 (43.9%)	7,876	2,892 (36.7%)	**< 0.001**
Age at registration	10,413	55.0 ± 11.7	2,537	52.9 ± 12.5	7,876	55.7 ± 11.3	**< 0.001**
Past or current history (%)							
Hypertension	9,739	1,562 (16.0%)	2,367	368 (15.6%)	7,372	1,194 (16.2%)	0.48
Dyslipidemia	9,687	436 (4.5%)	2,356	85 (3.6%)	7,331	351 (4.8%)	**0.02**
Diabetes mellitus	9,689	389 (4.0%)	2,356	105 (4.5%)	7,333	284 (3.9%)	0.21
Habits							
Smoking	9,859	3,492 (35.4%)	2,403	984 (41.0%)	7,456	2,508 (33.6%)	**< 0.001**
Habitual alcohol drinking	9,547	4,501 (47.1%)	2,336	1,252 (53.6%)	7,211	3,249 (45.1%)	**< 0.001**
Measurements							
Body length (cm)	9,986	155.0 ± 8.8	2,411	156.5 ± 9.1	7,575	154.5 ± 8.7	**< 0.001**
Body weight (kg)	9,989	55.6 ± 9.5	2,411	57.1 ± 10.1	7,578	55.1 ± 9.2	**< 0.001**
Body mass index (kg/m^2^)	9,986	23.1 ± 3.1	2,411	23.3 ± 3.2	7,575	23.0 ± 3.0	**0.005**
Systolic blood pressure (mmHg)	10,044	129.0 ± 20.9	2,427	128.7 ± 21.4	7,617	129.1 ± 20.7	0.15
Diastolic blood pressure (mmHg)	10,044	77.3 ± 12.3	2,427	77.5 ± 12.6	7,617	77.3 ± 12.1	0.73
Total cholesterol (mg/L)	10,403	191.7 ± 34.8	2,535	183.6 ± 34.3	7,868	194.3 ± 34.6	**< 0.001**
HDL cholesterol (mg/L)	10,403	51.3 ± 13.1	2,535	51.3 ± 13.9	7,868	51.3 ± 12.8	0.62
Lipoprotein(a) (mg/L)	10,413	204.1 ± 189.2	2,537	42.9 ± 19.8	7,876	256.0 ± 190.1	**< 0.001**
Triglyceride (mg/L)	10,402	115.9 ± 75.4	2,535	127.9 ± 93.2	7,867	112 ± 68.2	**< 0.001**
Blood glucose (mg/L)	10,400	103.3 ± 26.2	2,534	104.2 ± 28.7	7,866	103 ± 25.3	0.28
Hemoglobin A1c (%)	3,765	5.5 ± 0.7	985	5.5 ± 0.7	2,780	5.5 ± 0.7	0.45

Abbreviation: Lp(a), lipoprotein(a).

* Low Lp(a) group, Lp(a)<80 mg/L; intermediate-to-high Lp(a) group, Lp(a)≥80 mg/L.

† The p values for group comparison were calculated using the Mann-Whitney test or the Fisher exact test. Statistically significant p values are shown in boldface.

### Cox proportional hazards analysis

Table 2 shows the results of the proportional hazards analysis. A low Lp(a) level was a significant risk factor for all-cause deaths (p<0.001), cancer deaths (p = 0.003), and miscellaneous-cause deaths (p = 0.01). The hazard ratio (95% CI), 1.48 (1.15–1.92), for a low Lp(a) level for cancer deaths was almost the same as that (1.46, 1.00–2.13) for a male sex. A gender-specific analysis showed no significant differences in all-cause deaths or specific-cause deaths between genders (Table S2). We also estimated the risk of a low Lp(a) level for each primary site of malignancy (Table S3). A low Lp(a) level was a significant risk factor for liver cancer and cancers of the digestive system. The hazard ratio was very high (5.23; 95%CI, 2.01–13.6) for death from liver cancer. A low Lp(a) level was not a significant risk factor for death from lung cancer or other cancers. To exclude the possibility of reverse causation, 20 subjects with liver cirrhosis (n = 2) or liver cancer (n = 18) were excluded from the subjects. A subsequent Cox proportional hazard analysis showed similar results: the hazard ratio (95% CI) of a low Lp(a) level for death from cancer was 1.35 (1.03–1.77), p = 0.03. If the 91 subjects who died within two years after registration were excluded from the subjects, similar results were obtained: the hazard ratio for all-cause death was 1.43 (95%CI, 1.21–1.70; p<0.0001). When the subjects were divided into three Lp(a) groups, a very high Lp(a) level was not a risk factor for all-cause or any cause-specific deaths (Table S4).

**Table 2:** Cox proportional hazard analysis of low lipoproteinemia(a) for all-cause and cause-specific deaths (n = 10,413).

Variables	Hazard ratio (95% C.I.)	p value*
All-cause deaths		
Sex, men/women	1.56 (1.23–1.97)	**<0.001**
Age, per year	1.11 (1.10–1.11)	**<0.001**
Body mass index, per 1 kg/m^2^	0.98 (0.95–1.00)	0.07
Smoking history, yes/no	1.64 (1.32–2.05)	**<0.001**
Alcohol history, yes/no	1.10 (0.92–1.31)	0.31
Lp(a), low/intermediate-to-high group†	1.43 (1.21–1.68)	**<0.001**
Cardiovascular deaths		
Sex, men/women	1.21 (0.76–1.93)	0.41
Age, per year	1.13 (1.11–1.15)	**<0.001**
Body mass index, per 1 kg/m^2^	1.03 (0.98–1.08)	0.27
Smoking history, yes/no	1.55 (1.00–2.41)	0.05
Alcohol history, yes/no	1.26 (0.88–1.80)	0.21
Lp(a), low/intermediate-to-high group†	1.31 (0.93–1.84)	0.12
Cancer deaths		
Sex, men/women	1.46 (1.00–2.13)	**0.05**
Age, per year	1.09 (1.07–1.10)	**<0.001**
Body mass index, per 1 kg/m^2^	1.00 (0.97–1.05)	0.83
Smoking history, yes/no	2.04 (1.43–2.92)	**<0.001**
Alcohol history, yes/no	1.01 (0.77–1.34)	0.92
Lp(a), low/intermediate-to-high group†	1.48 (1.15–1.92)	**0.003**
Miscellaneous-cause deaths		
Sex, men/women	1.98 (1.34–2.91)	**<0.001**
Age, per year	1.11 (1.09–1.12)	**<0.001**
Body mass index, per 1 kg/m^2^	0.91 (0.87–0.95)	**<0.001**
Smoking history, yes/no	1.36 (0.95–1.95)	0.09
Alcohol history, yes/no	1.08 (0.81–1.45)	0.60
Lp(a), low/intermediate-to-high group†	1.45 (1.10–1.90)	**0.01**

Abbreviations: C.I., confidence interval, Lp(a), lipoprotein(a).

* Statistically significant p values are shown in boldface.

† Low Lp(a) group, Lp(a)<80 mg/L; intermediate-to-high Lp(a) group, Lp(a)≥80 mg/L.

## Discussion

The present study showed that a low Lp(a) level was a risk factor for all-cause, cancer, and miscellaneous-cause deaths. This is the first report describing the clinical and epidemiological significance of a low Lp(a) concentration. Since no definition of a low Lp(a) concentration, otherwise described as hypolipoproteinemia(a), exists, we defined an Lp(a) level of less than 80 mg/L as a low Lp(a) concentration in the present study. Further validation studies are necessary to determine the threshold of hypolipoproteinemia(a) in other populations.

### Lp(a) and all-cause/cardiovascular deaths

Although hyperlipoproteinemia(a) is an established risk factor for the onset of atherosclerotic disease, especially coronary artery disease and stroke [1]–[4], only a few reports have discussed the associations between hyperlipoproteinemia(a) and all-cause or cardiovascular deaths [32]–[34]. A meta-analysis of several long-term prospective studies of Caucasian subjects revealed that hyperlipoproteinemia(a) is a risk factor for coronary deaths, but not for cancer deaths or nonvascular deaths other than cancer [33]. The Chin-Shan Study showed that hyperlipoproteinemia(a) was a risk for all-cause deaths in a univariate analysis, but not in a multivariate analysis [34]. The present study showed that both a low and a very high Lp(a) level are risk factors for all-cause deaths. However, no cohort studies regarding hypolipoproteinemia(a) have been published to date.

### Lp(a) and longevity

Several reports regarding the association between Lp(a) and longevity have been made. Considering the cardiovascular risk associated with hyperlipoproteinemia(a), very elderly subjects were initially expected to have a low Lp(a) level. However, several studies have repeatedly reported the presence of high Lp(a) levels among centenarians [35]–[37]. These reports are consistent with our result that a low Lp(a) level was a risk factor for all-cause death.

Since a chronic inflammatory state persists in elderly people because of the appearance of autoimmunity and chronic inflammation of the upper respiratory and urinary tracts, and so on, the levels of inflammatory markers, such as CRP, IL6, and TNFα are likely to be elevated [35], [38]. As Lp(a) is an acute-phase reactant [39], the high Lp(a) level observed in centenarians might be ascribed to age-associated chronic inflammation.

### Lp(a) and cancer deaths

As an anti-neoplastic effect of apo(a) has been suggested, several case-controlled studies have been conducted regarding the association between the Lp(a) level and cancer. Patients with lung or breast cancer exhibit elevated Lp(a) levels [17]–[20], while the Lp(a) level was relatively low in patients with hepatocellular carcinoma [21,22]. No significant differences in the Lp(a) level were reported among patients with prostate cancer, ovarian cancer, or acute lymphoblastic lymphoma [23]–[25]. The present study showing the independent risk associated with a low Lp(a) level for cancer deaths is compatible with previous experimental studies.

A low Lp(a) level was correlated with higher cumulative death rates from liver cancer in this study. Retrospective studies of individuals who have received a hepatitis C virus-contaminated blood transfusion have shown that liver cirrhosis and liver cancer are likely to occur approximately 20 and 30–years after transfusion, respectively [40]. About 90% of cases of liver cancer in Japan are related to infection with hepatitis C or B virus [41]. Liver cirrhosis associated with liver cancer can lower the Lp(a) level as a result of liver dysfunction. Thus, reverse causation may explain some portion of the results; however, a low Lp(a) level was still a risk for all-cause, cancer, or miscellaneous-cause deaths even after the exclusion of subjects with liver cancer or liver cirrhosis or the exclusion of subjects who died within two years of registration. Overall, we assumed that the possibility of reverse causation was not large.

### Lp(a), miscellaneous-cause deaths, and inflammation

The association between a low Lp(a) level and miscellaneous-cause deaths was an unexpected result. The most common cause of miscellaneous-cause deaths was inflammatory disease, including pneumonia. Limited information is available regarding the association between the Lp(a) level and inflammation.

Lp(a) is an acute phase reactant [39]. The Lp(a) level increases by approximately twice the baseline level and peaks at 11 days after an acute myocardial infarction or at seven days after an operation, then returns to the baseline level after one month [39]. Compared with other acute phase reactants, such as CRP, α1-acid glycoprotein, α1-antitrypsin, or haptoglobin, the peak in the Lp(a) level is delayed, suggesting that Lp(a) may play a role in reducing inflammation or promoting tissue repair. As apo(a) is immunolocalized at small blood vessels in wounds [39], [42], it may be related to wound repair [43]. An experimental peritonitis model using apo(a) transgenic mice showed that apo(a) suppressed the inflammatory response by inhibiting neutrophil recruitment, suggesting that apo(a) is a cell-specific suppressor of the inflammatory response [44]. A clinical study of myocardial infarction reported that hyperlipoproteinemia(a) was associated with a decrease in coronary collateral circulation [45]. Thus, Lp(a) may also suppress angiogenesis in cases with inflammation. All these reports support the role of Lp(a) in tissue repair and the suppression of excess inflammation through the inhibition of angiogenesis or neutrophil recruitment. This anti-inflammatory and wound-repairing effect of Lp(a) is in line with our results regarding the significant association between a low Lp(a) level and miscellaneous-cause deaths. In any case, little is known about the relation between Lp(a) and inflammation, and future experimental studies are required.

### Limitations of this study

The contribution of Lp(a) to cardiovascular diseases differs among races [46], [47], but stratification was of minimal concern in the present study because all the subjects were Japanese.

Although the analysis of the low Lp(a) concentration had a sufficient statistical power, the relatively small number of deaths (1,072 participants) among the subjects seemed to limit the statistical power for detecting the hazard ratio of a very high Lp(a) concentration or primary site-specific cancer deaths.

Since serum Lp(a) levels are not significantly affected by external factors such as nutrition, smoking, drinking status, or the use of drugs [48] and are known to remain unchanged during long periods of life (12 years, or more than 20 years) [33, 49], we assumed that the Lp(a) data measured at the baseline examination would be representative of the Lp(a) throughout the study.

### Conclusions

This is the first report of the epidemiological importance of a low Lp(a) level for all-cause or cancer deaths. Further epidemiological studies are warranted to confirm our results. The present study seemed to support an anti-neoplastic effect of apo(a).

## Supporting Information

Figure S1 **Log-log survival plot for three causes of death.** The curves for cardiovascular deaths and miscellaneous-cause deaths are much higher than that for cancer deaths when the observation time is relatively short (early death); however, not surprisingly, the curve for cancer deaths becomes higher than the other two curves during later years. The close approximation of the three curves during later years provides evidence against the proportionality hypothesis.
(PPTX)

Figure S2 **Smoothed hazards functions with Kernel smoothing among three causes of death.** The smoothed hazard functions for miscellaneous-cause deaths and cardiovascular deaths are similar; however, this similarity is lost at eight to nine years after registration. The sharp increase in the hazard for miscellaneous-cause deaths after eight to nine years could arguably be disregarded because the standard errors increase for later observation times. As expected, the hazard for cancer deaths is much higher than that for cardiovascular deaths, and gradually increases with time.
(PPTX)

Figure S3 **Cumulative death rates for all-cause and cause-specific deaths among three lipoprotein(a) [Lp(a)] groups.** The cumulative death rates of the low Lp(a) group are significantly higher than those of the intermediate Lp(a) group for all-cause, cancer, and miscellaneous-cause deaths. The cumulative death rate of the very high Lp(a) group is not higher than that of the intermediate Lp(a).
(PPTX)

Figure S4 **Cumulative death rates for primary site-specific cancer deaths among two lipoprotein(a) [Lp(a)] groups.** The cumulative death rate of the low Lp(a) group [Lp(a)<80 mg/L] is significantly higher than that of the intermediate-to-high Lp(a) group [Lp(a)≥80 mg/L] in liver cancer and noncancerous causes.
(PPTX)

Table S1 **Cox proportional hazard analysis of serum lipoprotein(a) levels to cause-specific deaths.**
(DOC)

Table S2 **Gender-specific Cox proportional hazard analysis of low lipoproteinemia(a) for all-cause and cause-specific deaths.**
(DOC)

Table S3 **Cox proportional hazard analysis of low lipoproteinemia(a) for primary site-specific cancer deaths.**
(DOC)

Table S4 **Cox proportional hazard analysis of lipoproteinemia(a) for all-cause and cause-specific deaths [three ranks of lipoproteinemia(a)].**
(DOC)
