# Supplementary material for: Low Lipoprotein(a) Concentration Is Associated with Cancer and All-Cause Deaths: A Population-Based Cohort Study (The JMS Cohort Study)
Source: PLoS One. 2012 Apr 2;7(4):e31954. doi: 10.1371/journal.pone.0031954 (PMC3317664; doi:10.1371/journal.pone.0031954)
Supplement: Figure S1 — Log-log survival plot for three causes of death. The curves for cardiovascular deaths and miscellaneous-cause deaths are much higher than that for cancer deaths when the observation time is relatively short (early death); however, not surprisingly, the curve for cancer deaths becomes higher than the other two curves during later years. The close approximation of the three curves during later years provides evidence against the proportionality hypothesis. (PPTX) [file pone.0031954.s001.pptx]

## Slide 1
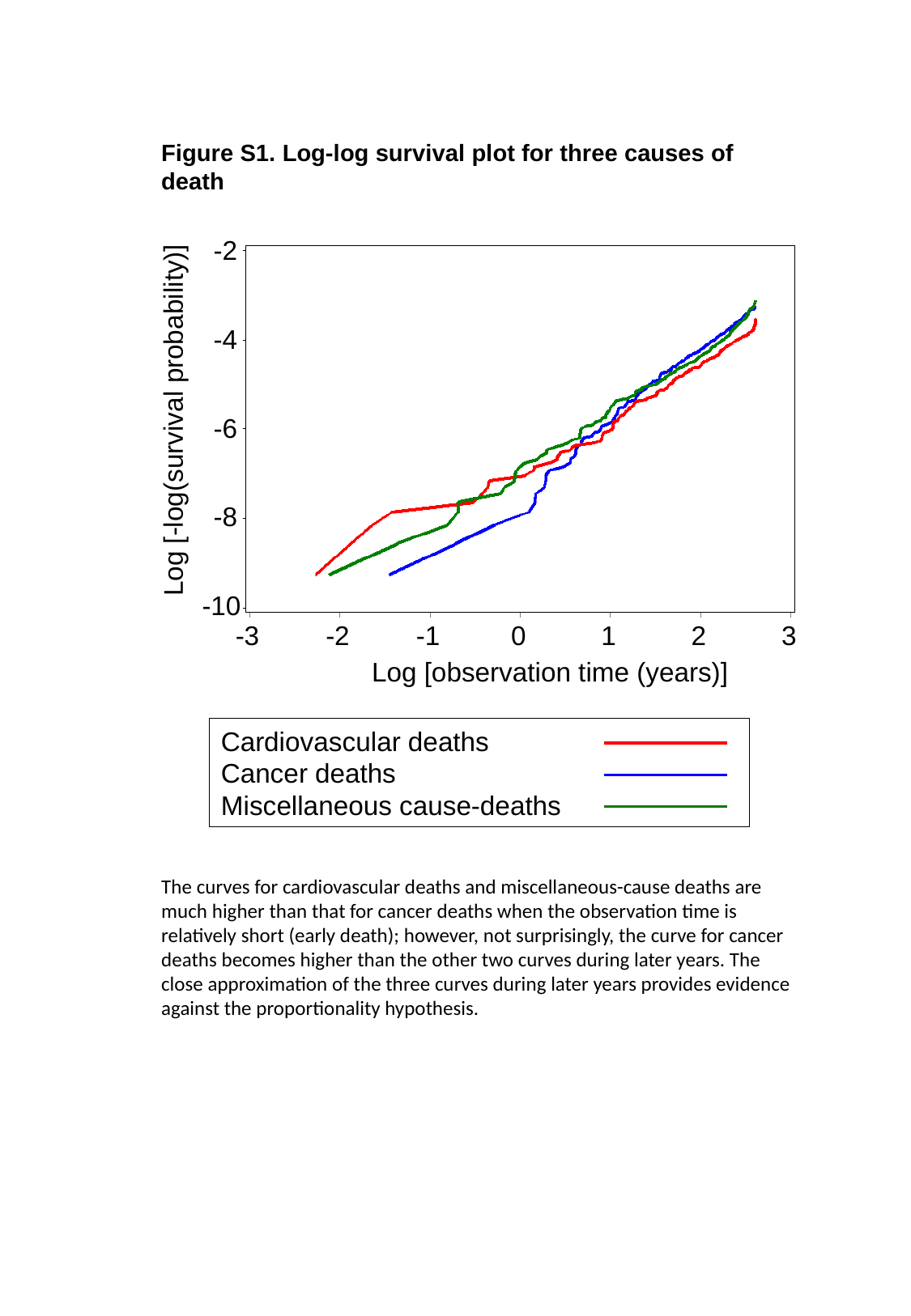

Figure S1. Log-log survival plot for three causes of death
-2
-4
Log [-log(survival probability)]
-6
-8
-10
-3
-2
-1
0
1
2
3
Log [observation time (years)]
Cardiovascular deaths
Cancer deaths
Miscellaneous cause-deaths
The curves for cardiovascular deaths and miscellaneous-cause deaths are much higher than that for cancer deaths when the observation time is relatively short (early death); however, not surprisingly, the curve for cancer deaths becomes higher than the other two curves during later years. The close approximation of the three curves during later years provides evidence against the proportionality hypothesis.
