# Supplementary material for: Low Lipoprotein(a) Concentration Is Associated with Cancer and All-Cause Deaths: A Population-Based Cohort Study (The JMS Cohort Study)
Source: PLoS One. 2012 Apr 2;7(4):e31954. doi: 10.1371/journal.pone.0031954 (PMC3317664; doi:10.1371/journal.pone.0031954)
Supplement: Figure S2 — Smoothed hazards functions with Kernel smoothing among three causes of death. The smoothed hazard functions for miscellaneous-cause deaths and cardiovascular deaths are similar; however, this similarity is lost at eight to nine years after registration. The sharp increase in the hazard for miscellaneous-cause deaths after eight to nine years could arguably be disregarded because the standard errors increase for later observation times. As expected, the hazard for cancer deaths is much higher than that for cardiovascular deaths, and gradually increases with time. (PPTX) [file pone.0031954.s002.pptx]

## Slide 1
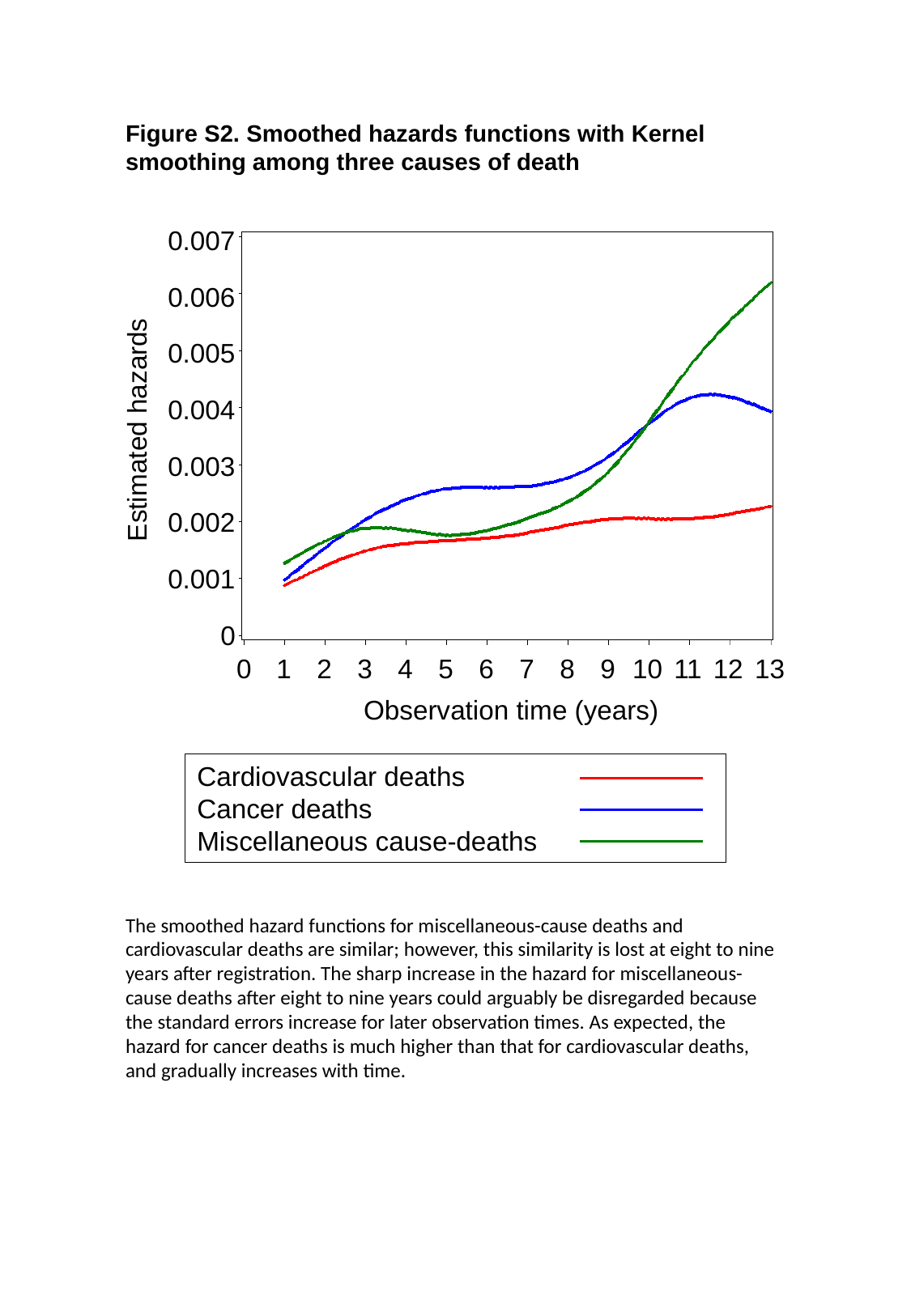

Figure S2. Smoothed hazards functions with Kernel smoothing among three causes of death
0.007
0.006
0.005
0.004
Estimated hazards
0.003
0.002
0.001
0
0
1
2
3
4
5
6
7
8
9
10
11
12
13
Observation time (years)
Cardiovascular deaths
Cancer deaths
Miscellaneous cause-deaths
The smoothed hazard functions for miscellaneous-cause deaths and cardiovascular deaths are similar; however, this similarity is lost at eight to nine years after registration. The sharp increase in the hazard for miscellaneous-cause deaths after eight to nine years could arguably be disregarded because the standard errors increase for later observation times. As expected, the hazard for cancer deaths is much higher than that for cardiovascular deaths, and gradually increases with time.
