# Supplementary material for: Low Lipoprotein(a) Concentration Is Associated with Cancer and All-Cause Deaths: A Population-Based Cohort Study (The JMS Cohort Study)
Source: PLoS One. 2012 Apr 2;7(4):e31954. doi: 10.1371/journal.pone.0031954 (PMC3317664; doi:10.1371/journal.pone.0031954)
Supplement: Figure S3 — Cumulative death rates for all-cause and cause-specific deaths among three lipoprotein(a) [Lp(a)] groups. The cumulative death rates of the low Lp(a) group are significantly higher than those of the intermediate Lp(a) group for all-cause, cancer, and miscellaneous-cause deaths. The cumulative death rate of the very high Lp(a) group is not higher than that of the intermediate Lp(a). (PPTX) [file pone.0031954.s003.pptx]

## Slide 1
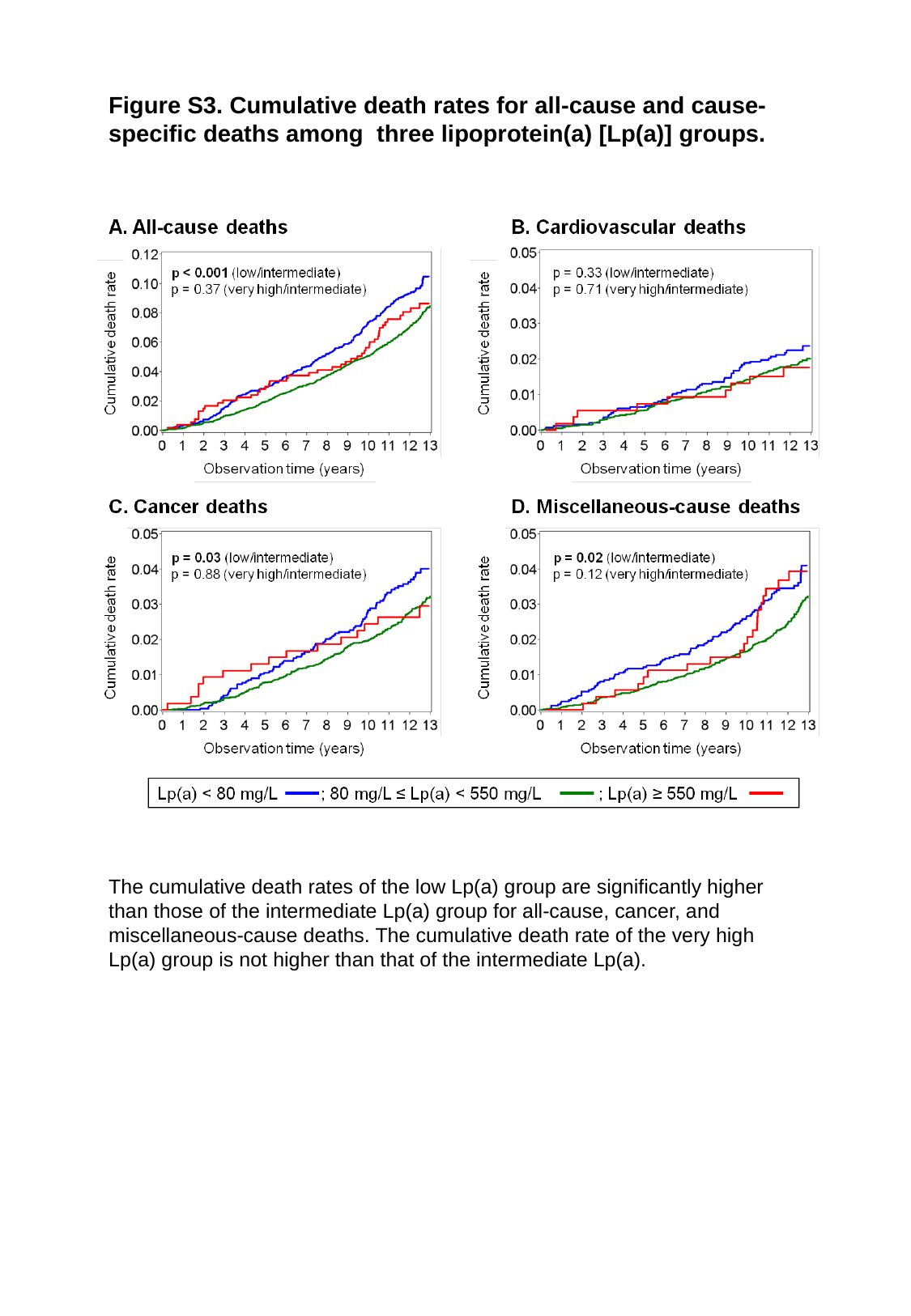

Figure S3. Cumulative death rates for all-cause and cause-specific deaths among three lipoprotein(a) [Lp(a)] groups.
The cumulative death rates of the low Lp(a) group are significantly higher than those of the intermediate Lp(a) group for all-cause, cancer, and miscellaneous-cause deaths. The cumulative death rate of the very high Lp(a) group is not higher than that of the intermediate Lp(a).
