# Supplementary material for: Low Lipoprotein(a) Concentration Is Associated with Cancer and All-Cause Deaths: A Population-Based Cohort Study (The JMS Cohort Study)
Source: PLoS One. 2012 Apr 2;7(4):e31954. doi: 10.1371/journal.pone.0031954 (PMC3317664; doi:10.1371/journal.pone.0031954)
Supplement: Figure S4 — Cumulative death rates for primary site-specific cancer deaths among two lipoprotein(a) [Lp(a)] groups. The cumulative death rate of the low Lp(a) group [Lp(a)<80 mg/L] is significantly higher than that of the intermediate-to-high Lp(a) group [Lp(a)≥80 mg/L] in liver cancer and noncancerous causes. (PPTX) [file pone.0031954.s004.pptx]

## Slide 1
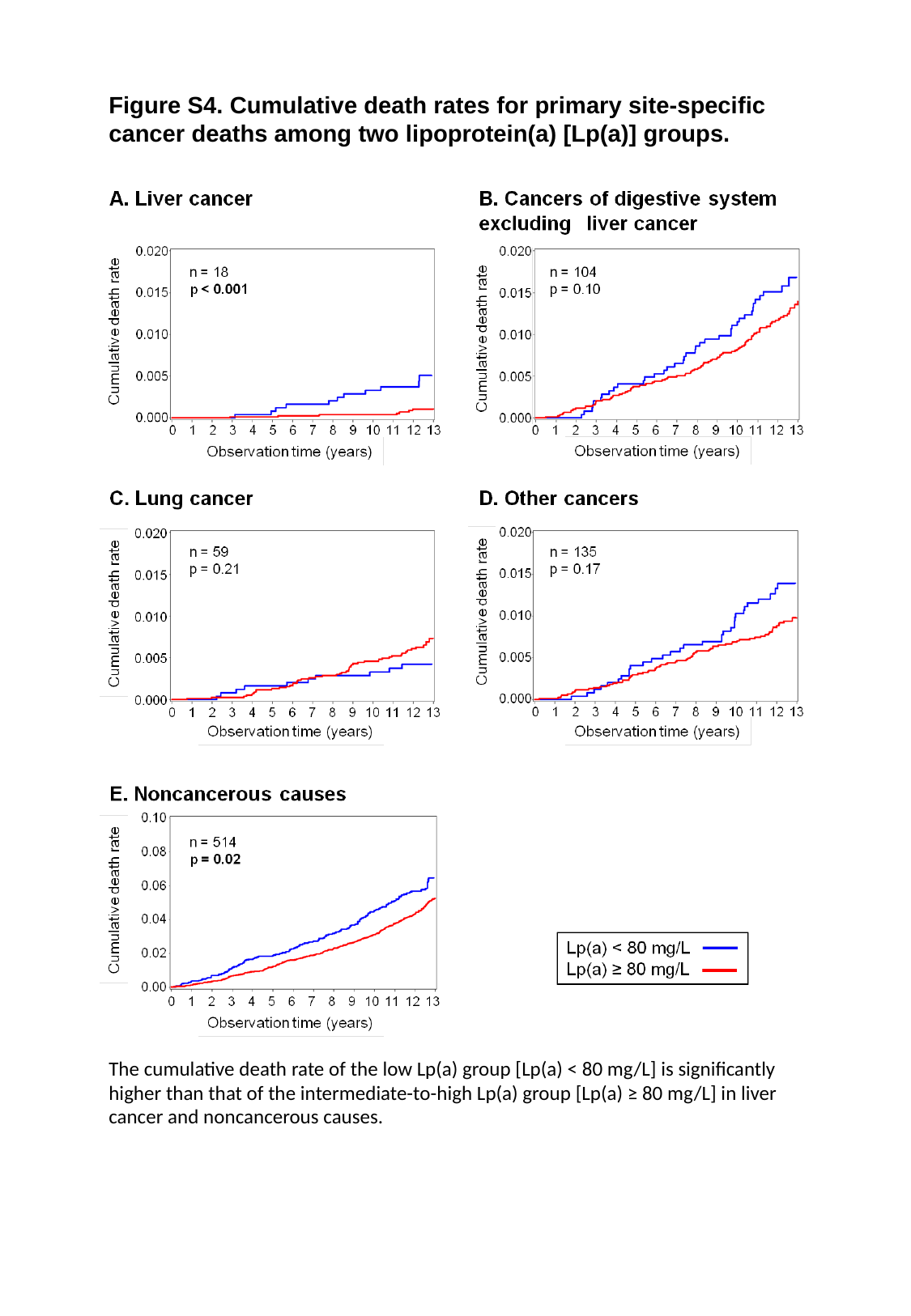

Figure S4. Cumulative death rates for primary site-specific cancer deaths among two lipoprotein(a) [Lp(a)] groups.
The cumulative death rate of the low Lp(a) group [Lp(a) < 80 mg/L] is significantly higher than that of the intermediate-to-high Lp(a) group [Lp(a) ≥ 80 mg/L] in liver cancer and noncancerous causes.
