# Supplementary material for: Low Lipoprotein(a) Concentration Is Associated with Cancer and All-Cause Deaths: A Population-Based Cohort Study (The JMS Cohort Study)
Source: PLoS One. 2012 Apr 2;7(4):e31954. doi: 10.1371/journal.pone.0031954 (PMC3317664; doi:10.1371/journal.pone.0031954)
Supplement: Table S1 — Cox proportional hazard analysis of serum lipoprotein(a) levels to cause-specific deaths. (DOC) [file pone.0031954.s005.doc]

**Table S1. Cox proportional hazard analysis of serum lipoprotein(a) levels to cause-specific deaths**

|  | Lp(a) level | Hazard ratio (95% C.I.) | *P* value* |
| --- | --- | --- | --- |
| Cancer death | |  |  |
|  | Q1/Q2 | 1.29 (0.94 - 1.78) | 0.12 |
|  | Q1/Q3 | 1.51 (1.10 - 2.09) | **0.01** |
|  | Q1/Q4 | 1.67 (1.20 - 2.33) | **0.003** |
|  | Q2/Q3 | 1.17 (0.84 - 1.64) | 0.35 |
|  | Q2/Q4 | 1.29 (0.91 - 1.83) | 0.15 |
|  | Q3/Q4 | 1.10 (0.78 - 1.56) | 0.58 |
| Cardiovascular death | |  |  |
|  | Q1/Q2 | 1.00 (0.67 - 1.51) | 0.99 |
|  | Q1/Q3 | 1.64 (1.06 - 2.54) | **0.03** |
|  | Q1/Q4 | 1.38 (0.90 - 2.10) | 0.14 |
|  | Q2/Q3 | 1.64 (1.06 - 2.53) | **0.03** |
|  | Q2/Q4 | 1.37 (0.90 - 2.09) | 0.14 |
|  | Q3/Q4 | 0.84 (0.54 - 1.31) | 0.43 |
| Miscellaneous-cause death | |  |  |
|  | Q1/Q2 | 1.34 (0.95 - 1.91) | 0.10 |
|  | Q1/Q3 | 1.42 (1.02 - 1.98) | **0.04** |
|  | Q1/Q4 | 1.58 (1.12 - 2.23) | **0.01** |
|  | Q2/Q3 | 1.05 (0.74 - 1.51) | 0.77 |
|  | Q2/Q4 | 1.17 (0.81 - 1.70) | 0.39 |
|  | Q3/Q4 | 1.11 (0.79 - 1.58) | 0.54 |

Abbreviations: C.I., confidence interval; Lp(a), lipoprotein(a); Q1, the first quarter [Lp(a) < 80 mg/L]; Q2, the second quarter [80 ≤ Lp(a) < 150]; Q3, the third quarter [150 ≤ Lp(a) < 270]; Q4, the fourth quarter [270 ≤ Lp(a)]

This analysis was adjusted for age, sex, body mass index, and smoking and habitual drinking histories.

*Statistically significant *P* values are shown in boldface.
