# Supplementary material for: Low Lipoprotein(a) Concentration Is Associated with Cancer and All-Cause Deaths: A Population-Based Cohort Study (The JMS Cohort Study)
Source: PLoS One. 2012 Apr 2;7(4):e31954. doi: 10.1371/journal.pone.0031954 (PMC3317664; doi:10.1371/journal.pone.0031954)
Supplement: Table S2 — Gender-specific Cox proportional hazard analysis of low lipoproteinemia(a) for all-cause and cause-specific deaths. (DOC) [file pone.0031954.s006.doc]

**Table S2. Gender-specific Cox proportional hazard analysis of low lipoproteinemia(a) for all-cause and cause-specific deaths**

|  |  | Men (n = 4,005) |  |  | Women (n = 6,408) |  |
| --- | --- | --- | --- | --- | --- | --- |
|  | Variables | Hazard ratio (95% C.I.) | *P* value* |  | Hazard ratio (95% C.I.) | *P* value* |
| All-cause deaths | |  |  |  |  |  |
|  | Age, per year | 1.10 (1.08 - 1.11) | **< 0.001** |  | 1.12 (1.11 - 1.14) | **< 0.001** |
|  | Body mass index, per 1 kg/m^2^ | 0.96 (0.93 - 1.00) | **0.04** |  | 0.99 (0.95 - 1.03) | 0.61 |
|  | Smoking history, yes/no | 1.80 (1.36 - 2.39) | **< 0.001** |  | 1.28 (0.84 - 1.94) | 0.25 |
|  | Alcohol history, yes/no | 1.06 (0.85 - 1.33) | 0.62 |  | 1.15 (0.87 - 1.52) | 0.31 |
|  | Lp(a), low/intermediate-to-high group^†^ | 1.38 (1.12 - 1.69) | **0.003** |  | 1.51 (1.16 - 1.97) | **0.003** |
| Cardiovascular deaths | |  |  |  |  |  |
|  | Age, per year | 1.10 (1.08 - 1.13) | **< 0.001** |  | 1.10 (1.08 - 1.13) | **< 0.001** |
|  | Body mass index, per 1 kg/m^2^ | 1.03 (0.96 - 1.11) | 0.42 |  | 1.03 (0.96 - 1.11) | 0.42 |
|  | Smoking history, yes/no | 1.66 (0.94 - 2.95) | 0.08 |  | 1.66 (0.94 - 2.95) | 0.72 |
|  | Alcohol history, yes/no | 1.15 (0.71 - 1.88) | 0.57 |  | 1.15 (0.71 - 1.88) | 0.19 |
|  | Lp(a), low/intermediate-to-high group^†^ | 1.12 (0.71 - 1.77) | 0.64 |  | 1.12 (0.71 - 1.77) | 0.10 |
| Cancer deaths | |  |  |  |  |  |
|  | Age, per year | 1.08 (1.07 - 1.10) | **< 0.001** |  | 1.09 (1.07 - 1.12) | **< 0.001** |
|  | Body mass index, per 1 kg/m^2^ | 0.99 (0.94 - 1.04) | 0.68 |  | 1.02 (0.96 - 1.09) | 0.45 |
|  | Smoking history, yes/no | 2.40 (1.47 - 3.93) | **0.001** |  | 1.54 (0.82 - 2.89) | 0.18 |
|  | Alcohol history, yes/no | 0.95 (0.67 - 1.35) | 0.77 |  | 1.14 (0.73 - 1.77) | 0.56 |
|  | Lp(a), low/intermediate-to-high group^†^ | 1.40 (1.01 - 1.94) | **0.04** |  | 1.65 (1.09 - 2.51) | **0.02** |
| Miscellaneous-cause deaths | |  |  |  |  |  |
|  | Age, per year | 1.10 (1.08 - 1.12) | **< 0.001** |  | 1.12 (1.09 - 1.14) | **< 0.001** |
|  | Body mass index, per 1 kg/m^2^ | 0.90 (0.85 - 0.95) | **0.001** |  | 0.92 (0.86 - 0.99) | **0.02** |
|  | Smoking history, yes/no | 1.46 (0.95 - 2.25) | 0.09 |  | 1.06 (0.48 - 2.30) | 0.89 |
|  | Alcohol history, yes/no | 1.14 (0.79 - 1.66) | 0.49 |  | 0.98 (0.59 - 1.62) | 0.94 |
|  | Lp(a), low/intermediate-to-high group^†^ | 1.52 (1.08 - 2.12) | **0.02** |  | 1.31 (0.81 - 2.12) | 0.27 |

Abbreviations: C.I., confidence interval, Lp(a), lipoprotein(a)

*Statistically significant *P* values are shown in boldface.

^†^Low Lp(a) group, Lp(a) < 80 mg/L; intermediate-to-high Lp(a) group, Lp(a) ≥ 80 mg/L
