# Supplementary material for: Low Lipoprotein(a) Concentration Is Associated with Cancer and All-Cause Deaths: A Population-Based Cohort Study (The JMS Cohort Study)
Source: PLoS One. 2012 Apr 2;7(4):e31954. doi: 10.1371/journal.pone.0031954 (PMC3317664; doi:10.1371/journal.pone.0031954)
Supplement: Table S3 — Cox proportional hazard analysis of low lipoproteinemia(a) for primary site-specific cancer deaths. (DOC) [file pone.0031954.s007.doc]

**Table S3. Cox proportional hazard analysis of low lipoproteinemia(a) for primary site-specific cancer deaths**

|  | Variables | Hazard ratio (95% C.I.) | *P* value* |
| --- | --- | --- | --- |
| Liver cancer | |  |  |
|  | Sex, men/women | 14.5 (1.41 - 149) | **0.02** |
|  | Age, per year | 1.08 (1.03 - 1.14) | **0.002** |
|  | Body mass index, per 1 kg/m^2^ | 0.94 (0.79 - 1.11) | 0.46 |
|  | Smoking history, yes/no | 1.77 (0.43 - 7.26) | 0.43 |
|  | Alcohol history, yes/no | 1.18 (0.37 - 3.84) | 0.78 |
|  | Lp(a), low/intermediate-to-high group^†^ | 5.23 (2.01 - 13.6) | **< 0.001** |
| Cancers of digestive system excluding liver cancer | | |  |
|  | Sex, men/women | 0.89 (0.46 - 1.72) | 0.74 |
|  | Age, per year | 1.10 (1.08 - 1.13) | **< 0.001** |
|  | Body mass index, per 1 kg/m^2^ | 0.99 (0.92 - 1.06) | 0.69 |
|  | Smoking history, yes/no | 2.20 (1.17 - 4.14) | **0.01** |
|  | Alcohol history, yes/no | 0.89 (0.54 - 1.45) | 0.63 |
|  | Lp(a), low/intermediate-to-high group^†^ | 1.75 (1.12 - 2.73) | **0.01** |
| Lung cancer | |  |  |
|  | Sex, men/women | 2.23 (0.85 - 5.86) | 0.10 |
|  | Age, per year | 1.10 (1.06 - 1.13) | **< 0.001** |
|  | Body mass index, per 1 kg/m^2^ | 0.97 (0.88 - 1.07) | 0.59 |
|  | Smoking history, yes/no | 4.37 (1.71 - 11.2) | **0.002** |
|  | Alcohol history, yes/no | 0.84 (0.45 - 1.56) | 0.57 |
|  | Lp(a), low/intermediate-to-high group^†^ | 0.70 (0.34 - 1.43) | 0.33 |
| Other cancers | |  |  |
|  | Sex, men/women | 1.48 (0.84 - 2.60) | 0.17 |
|  | Age, per year | 1.07 (1.05 - 1.09) | **< 0.001** |
|  | Body mass index, per 1 kg/m^2^ | 1.04 (0.98 - 1.11) | 0.19 |
|  | Smoking history, yes/no | 1.48 (0.87 - 2.52) | 0.15 |
|  | Alcohol history, yes/no | 1.20 (0.78 - 1.85) | 0.41 |
|  | Lp(a), low/intermediate-to-high group^†^ | 1.40 (0.94 - 2.08) | 0.10 |
| Other noncancerous causes | |  |  |
|  | Sex, men/women | 1.61 (1.20 - 2.17) | **0.002** |
|  | Age, per year | 1.12 (1.10 - 1.13) | **< 0.001** |
|  | Body mass index, per 1 kg/m^2^ | 0.96 (0.93 - 0.99) | **0.01** |
|  | Smoking history, yes/no | 1.44 (1.09 - 1.90) | **0.01** |
|  | Alcohol history, yes/no | 1.15 (0.92 - 1.44) | 0.23 |
|  | Lp(a), low/intermediate-to-high group^†^ | 1.39 (1.12 - 1.72) | **0.002** |

Abbreviations: C.I., confidence interval; Lp(a), lipoprotein(a)

*Statistically significant *P* values are shown in boldface.

^†^Low Lp(a) group, Lp(a) < 80 mg/L; intermediate-to-high Lp(a) group, Lp(a) ≥ 80 mg/L.
