# Supplementary material for: Low Lipoprotein(a) Concentration Is Associated with Cancer and All-Cause Deaths: A Population-Based Cohort Study (The JMS Cohort Study)
Source: PLoS One. 2012 Apr 2;7(4):e31954. doi: 10.1371/journal.pone.0031954 (PMC3317664; doi:10.1371/journal.pone.0031954)
Supplement: Table S4 — Cox proportional hazard analysis of lipoproteinemia(a) for all-cause and cause-specific deaths [three ranks of lipoproteinemia(a)]. (DOC) [file pone.0031954.s008.doc]

**Table S4. Cox proportional hazard analysis of lipoproteinemia(a) for all-cause and cause-specific deaths [three ranks of lipoproteinemia(a)]**

|  | Variables | Hazard ratio  (95% C.I.) | *P* values |
| --- | --- | --- | --- |
| All-cause deaths | |  |  |
|  | Sex, men/women | 1.55 (1.23 - 1.96) | **< 0.001** |
|  | Age, per year | 1.11 (1.10 - 1.11) | **< 0.001** |
|  | Body mass index, per 1 kg/m^2^ | 0.98 (0.95 - 1.00) | 0.06 |
|  | Smoking history, yes/no | 1.64 (1.32 - 2.04) | **< 0.001** |
|  | Alcohol history, yes/no | 1.10 (0.92 - 1.31) | 0.31 |
|  | Lp(a), low/intermediate group | 1.39 (1.17 - 1.65) | **< 0.001** |
|  | Lp(a), very high/intermediate group | 0.91 (0.75 - 1.10) | 0.32 |
| Cardiovascular deaths | |  |  |
|  | Sex, men/women | 1.20 (0.76 - 1.91) | 0.43 |
|  | Age, per year | 1.13 (1.11 - 1.15) | **< 0.001** |
|  | Body mass index, per 1 kg/m^2^ | 1.03 (0.98 - 1.08) | 0.30 |
|  | Smoking history, yes/no | 1.55 (1.00 - 2.40) | 0.05 |
|  | Alcohol history, yes/no | 1.26 (0.88 - 1.80) | 0.20 |
|  | Lp(a), low/intermediate group | 1.24 (0.87 - 1.77) | 0.23 |
|  | Lp(a), very high/intermediate group | 0.82 (0.55 - 1.23) | 0.34 |
| Cancer deaths | |  |  |
|  | Sex, men/women | 1.46 (1.00 - 2.12) | 0.05 |
|  | Age, per year | 1.09 (1.07 - 1.10) | **< 0.001** |
|  | Body mass index, per 1 kg/m^2^ | 1.00 (0.96 - 1.05) | 0.86 |
|  | Smoking history, yes/no | 2.04 (1.42 - 2.92) | **< 0.001** |
|  | Alcohol history, yes/no | 1.02 (0.77 - 1.34) | 0.92 |
|  | Lp(a), low/intermediate group | 1.44 (1.10 - 1.89) | **0.008** |
|  | Lp(a), very high/intermediate group | 0.90 (0.65 - 1.23) | 0.49 |
| Miscellaneous-cause deaths | |  |  |
|  | Sex, men/women | 1.98 (1.34 - 2.91) | **< 0.001** |
|  | Age, per year | 1.11 (1.09 - 1.12) | **< 0.001** |
|  | Body mass index, per 1 kg/m^2^ | 0.91 (0.87 - 0.95) | **< 0.001** |
|  | Smoking history, yes/no | 1.36 (0.95 - 1.95) | 0.09 |
|  | Alcohol history, yes/no | 1.08 (0.81 - 1.45) | 0.60 |
|  | Lp(a), low/intermediate group | 1.44 (1.08 - 1.92) | **0.01** |
|  | Lp(a), very high/intermediate group | 0.98 (0.72 - 1.35) | 0.92 |

C.I., confidence interval; Lp(a), lipoprotein(a)

Low Lp(a) group, Lp(a) < 80 mg/L; intermediate Lp(a) group, 80 mg/L ≤ Lp(a) < 550 mg/L; and very high Lp(a) group, Lp(a) ≥ 550 mg/L

Statistically significant *P* values are shown in boldface.
